# Simultaneous Bilateral Central Retinal Artery Occlusions: Partial Recovery With Multimodal Therapy

**DOI:** 10.7759/cureus.104600

**Published:** 2026-03-03

**Authors:** Jessie R Cai, Glen C Hawkins, Xi Jia, Alex Newton, Ian C Francis

**Affiliations:** 1 Department of Neurology, Prince of Wales Hospital, Sydney, AUS; 2 Faculty of Medicine, University of New South Wales, Sydney, AUS; 3 Department of Hyperbaric Medicine, Prince of Wales Hospital, Sydney, AUS; 4 Department of Neurology, St George Hospital, Sydney, AUS; 5 Department of Emergency Medicine, Prince of Wales Hospital, Sydney, AUS; 6 Department of Ophthalmology, Prince of Wales Hospital, Sydney, AUS; 7 Department of Ophthalmology, Northern Beaches Hospital, Sydney, AUS

**Keywords:** acute retinal stroke, acute vision loss, central retinal artery occlusion (crao), dabigatran reversal, giant cell arteritis (gca), hyperbaric oxygen therapy (hbot), intravenous thrombolysis, ocular neovascularization, retinal ischaemia, systemic thrombolysis

## Abstract

Central retinal artery occlusion (CRAO) is a rare cause of sudden, profound vision loss, and bilateral involvement is exceptionally uncommon, with no clearly established optimal management. We describe a patient who developed near-simultaneous, painless vision loss in both eyes and underwent urgent multidisciplinary assessment followed by systemic and ocular therapies intended to restore perfusion and limit ischemic injury. Although partial visual recovery was achieved, the course was complicated by retinal neovascularization requiring treatment and resulting in severe long-term visual impairment. This case underscores the diagnostic urgency, therapeutic uncertainty, and importance of close longitudinal follow-up in bilateral CRAO.

## Introduction

Central retinal artery occlusion (CRAO) is an ophthalmic emergency as it represents an acute ischemic stroke of the retina. It typically causes sudden, painless vision loss and is associated with a poor visual prognosis [1]. In most individuals, the first branch of the internal carotid artery is the ophthalmic artery, which gives rise to the central retinal artery. This vessel supplies the inner retina. In the majority of cases, the central retinal artery also supplies the fovea, which is critical for central vision. However, in approximately one-third of individuals, a cilioretinal artery, arising from the posterior ciliary circulation, supplies the fovea instead. In such cases, CRAO may result in less severe visual loss [2].

The estimated incidence of CRAO is approximately two per 100,000 annually [2,3]. By contrast, bilateral CRAO is exceedingly rare, occurring in fewer than 2% of cases [4]. Despite its profound functional consequences, no standardized treatment guidelines exist for either unilateral or bilateral CRAO due to a paucity of robust efficacy data [2]. Current management strategies include intravenous thrombolysis (IVT), intraocular pressure (IOP)-lowering interventions, and, in selected cases, hyperbaric oxygen therapy (HBOT) [2].

A recent randomized, controlled, phase III trial comparing early IVT with oral aspirin, in patients treated within a mean time of less than four hours from symptom onset, found no significant difference in visual acuity improvement at one month [5]. Nevertheless, observational data suggest that IVT administered within an acute treatment window may be both beneficial and safe [6,7], and ongoing randomized controlled trials are evaluating early IVT use in CRAO [8,9].

This report describes the management of an exceedingly rare case of simultaneous bilateral CRAO and documents the extensive investigations undertaken to determine its etiology. We aim to provide insight into potential treatment outcomes and treatment-related adverse effects. Given the absence of standardized treatment guidelines, management must remain individualized, depending on patient characteristics and local resource availability.

This article was previously presented as a meeting abstract for the ANZAN Annual Scientific Meeting in May 2024 and as a poster for the RANZCO Annual Scientific Meeting on November 16, 2025.

## Case presentation

A 68-year-old man with a history of atrial fibrillation treated with dabigatran etexilate (Pradaxa), hypertrophic cardiomyopathy, hypertension, and type 2 diabetes mellitus presented to the emergency department 90 minutes after sequential, painless, complete vision loss, initially in the right eye, followed by the left eye within five minutes. He reported no other symptoms suggestive of giant cell arteritis (GCA). He had otherwise been well.

Visual acuity was no light perception bilaterally, and the pupils were dilated and nonreactive. IOPs were normal at 18 mmHg in the right eye and 16 mmHg in the left. Fundus examination revealed bilateral retinal pallor with Hollenhorst plaques (cholesterol emboli) in the retinal arteries. In the right eye, cilioretinal artery sparing was present, demonstrated by a localized area of preserved retina temporal to the optic disc. No cilioretinal sparing was observed in the left eye, which showed diffuse retinal edema. No other focal neurological deficits were identified.

Urgent hyperacute stroke imaging with computed tomography (CT) of the brain and angiography of the aortic arch to the Circle of Willis demonstrated no acute infarction or large-vessel occlusion. The patient’s full blood count was normal, except for a mild microcytic, hypochromic anemia. Renal and hepatic function tests were within normal limits. Inflammatory markers (CRP and ESR) were mildly elevated. The patient’s coagulation profile was elevated (Table 1), consistent with their recent dabigatran use. To permit safe systemic thrombolysis, dabigatran reversal with 5 g idarucizumab (Praxbind) was administered 3 hours and 20 minutes after symptom onset.

**Table 1:** Relevant laboratory results at presentation

Test	Value	Interpretation	Reference range
Hemoglobin	127 g/L	Decreased	130-180 g/L
Mean corpuscular volume	66 fL	Decreased	80-100 fL
Mean corpuscular hemoglobin	20.3 pg	Decreased	26.5-033 pg
Platelets	208 x 10^9^/L	Within normal limits	150-450 x 10^9^/L
Creatinine	78 μmol/L	Within normal limits	60-110 μmol/L
Estimated glomerular filtration rate	86 mL/min/1.73 m^2^	Within normal limits	>90 mL/min/1.73 m^2^
Alkaline phosphatase	100 U/L	Within normal limits	30-110 U/L
Prothrombin time	15.6 seconds	Elevated	9-13 seconds
International normalized ratio	1.4	Elevated	0.8-1.3
Activated partial thromboplastin time	59.9 seconds	Elevated	25-37 seconds
Erythrocyte sedimentation rate (ESR)	18 mm/h	Elevated	0-14 mm/h
C-reactive protein (CRP)	7 mg/L	Elevated	<3 mg/L
Glycated hemoglobin (HbA1c)	6.8%	Elevated	4%-6%
Total cholesterol	3.6 mmol/L	Within normal limits	<5.6 mmol/L
Triglycerides	1.1 mmol/L	Within normal limits	<2.1 mmol/L
High-density lipoprotein cholesterol	1.3 mmol/L	Within normal limits	>0.9 mmol/L
Low-density lipoprotein cholesterol	1.8 mmol/L	Within normal limits	<3.1 mmol/L

IVT with alteplase (0.9 mg/kg) was administered four hours after symptom onset. Bilateral anterior chamber paracenteses were performed seven hours after onset, reducing IOP to 4-6 mmHg, from 18 mmHg in the right eye and 16 mmHg in the left at presentation. Given the catastrophic implications of bilateral blindness, high-dose intravenous methylprednisolone (1,000 mg daily) was initiated empirically on the day of presentation. Oral acetazolamide (Diamox) 250 mg twice a day was commenced to reduce IOP further. It was discontinued after two weeks because of worsening renal impairment.

The hyperbaric medicine team at nearby Prince of Wales Hospital (Sydney, Australia) was consulted. Due to transfer logistics, hyperbaric treatment commenced 22 hours after symptom onset. Two initial sessions were delivered at 284 kPa, with 100% oxygen for 120 minutes each, separated by a three-hour interval, followed by a third session at 243 kPa for 90 minutes the next day. The planned five-session course was not completed because of barotrauma-related otalgia.

**Figure 1:**
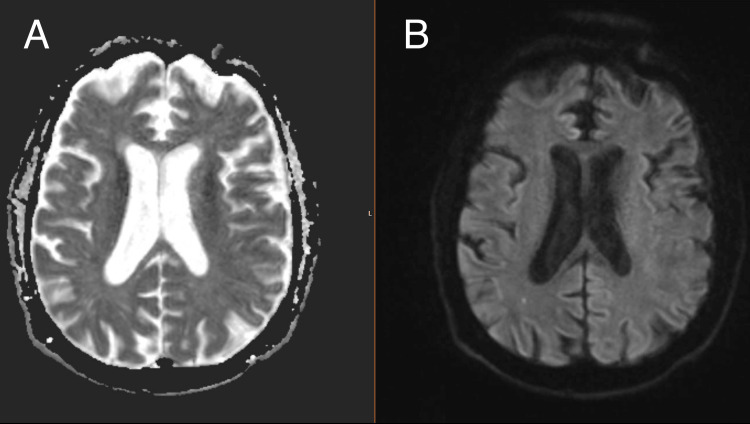
Magnetic resonance imaging of the brain demonstrating subacute embolic infarcts (A) Apparent diffusion coefficient (ADC) sequence demonstrating no corresponding abnormal signal in the right parietal lobe. (B) Diffusion-weighted imaging (DWI) sequence demonstrating a small hyperintense focus in the right parietal lobe. The presence of DWI hyperintensity without a corresponding abnormality on ADC is consistent with a subacute infarct in the right parietal lobe.

Extensive serologic testing and lumbar puncture with cerebrospinal fluid analysis revealed no autoimmune, infectious, or vasculitic etiology. Whole-body F-fluorodeoxyglucose positron emission tomography/computed tomography (F-FDG PET-CT) and temporal artery biopsy performed four and five days after steroid initiation showed no evidence of GCA. Brain magnetic resonance imaging (MRI) revealed small embolic infarcts in the right parietal lobe (Figure 1) and cerebellum. Carotid ultrasonography showed no significant stenosis or occlusion. A transesophageal echocardiogram did not demonstrate an intracardiac source of embolus. Thrombi, structural abnormalities in the heart, and valvular pathology were excluded.

**Figure 2:**
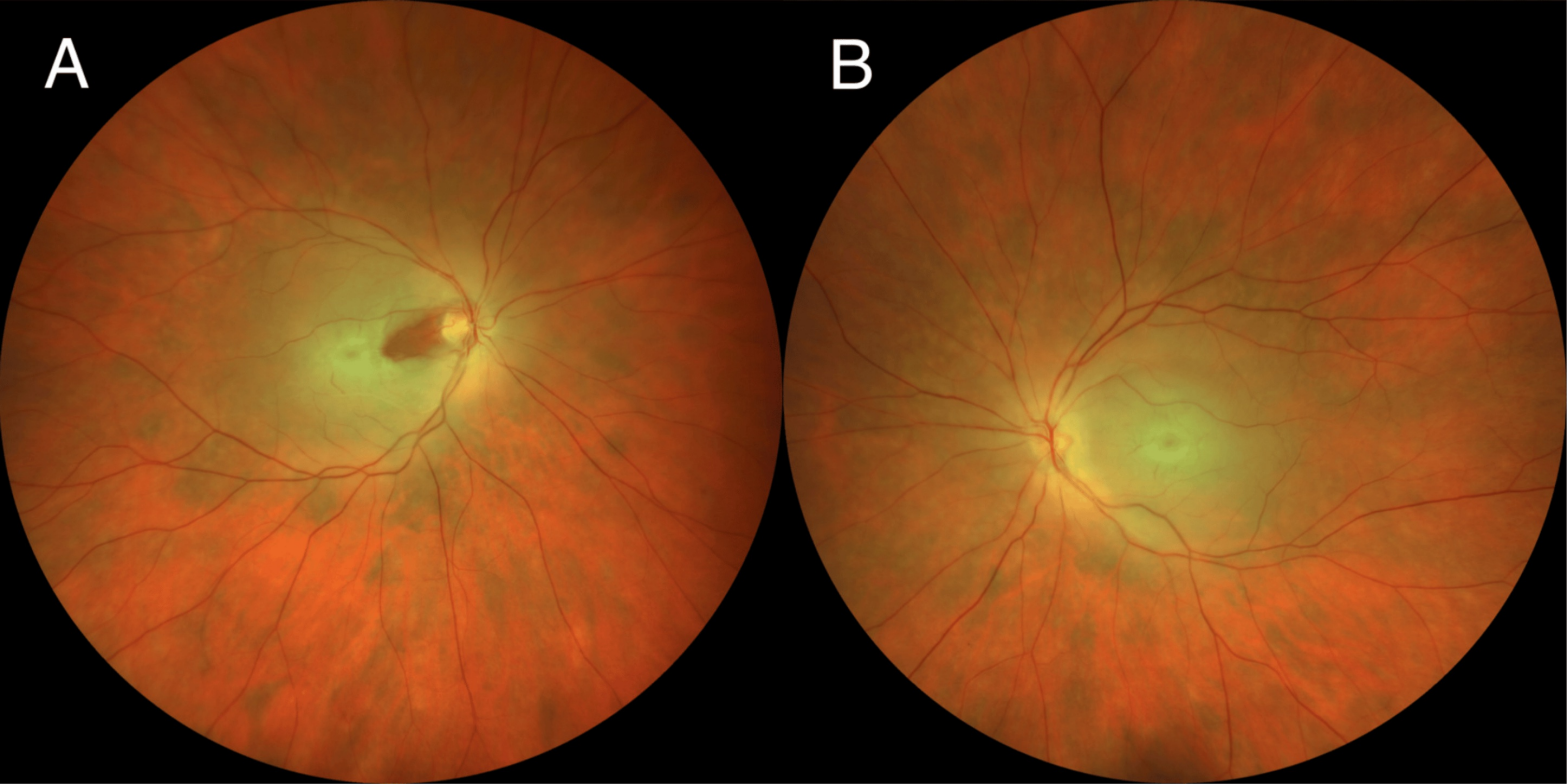
Color fundus photographs obtained three days after the onset of bilateral central retinal artery occlusions (A) The right fundus demonstrates extensive macular edema with preservation of the retina temporal to the optic disc due to cilioretinal artery sparing. (B) The left fundus demonstrates diffuse macular edema due to the absence of cilioretinal circulation. Bilateral cherry-red spots are present.

Three days after admission, fundus imaging demonstrated retinal edema encroaching on the fovea in the right eye, with persistent diffuse edema throughout the left retina (Figure 2). Visual acuity improved to hand-movement perception bilaterally at this point. By day 19, the patient was able to see counting fingers. At one month following admission, bilateral preretinal hemorrhages and retinal neovascularization developed, necessitating panretinal photocoagulation. Ten months later, vision stabilized at light perception in the right eye and counting fingers in the left.

## Discussion

This report illustrates the rare occurrence of near-simultaneous bilateral CRAO. This was most likely secondary to cholesterol emboli, given the observation of bilateral Hollenhorst plaques at presentation [4]. Although carotid atherosclerosis is the most common embolic source [4], the absence of significant carotid occlusive disease suggested a cardiac origin, although CT angiography of the aortic arch and transesophageal echocardiography did not reveal definite cardiac pathology. In the absence of formal treatment guidelines from major stroke organizations [2], multiple interventions were attempted, including IVT, corticosteroids, IOP reduction with acetazolamide and anterior chamber paracenteses, and HBOT.

IVT, the cornerstone of acute ischemic stroke management, has been cautiously extrapolated to CRAO management [2]. Meta-analyses suggest that thrombolysis within 4.5 hours may improve visual outcomes [2], while experimental studies indicate irreversible retinal injury may occur within four hours of occlusion [10]. However, robust safety and efficacy data remain limited, particularly in anticoagulated patients [5,11]. Indeed, this case reports the first use of dabigatran reversal followed by IVT in bilateral CRAO. Reversal of anticoagulation was required, given the patient’s recent dabigatran use, consistent with safety recommendations [12]. Notably, the incidence of ischemic or hemorrhagic stroke in anticoagulated atrial fibrillation patients receiving dabigatran is approximately 0.63 per 100 person-years [13], underscoring the exceptional rarity of this presentation. 

Given the low likelihood of bilateral CRAO in an adequately anticoagulated patient, extensive investigations were undertaken to exclude inflammatory or autoimmune causes. Negative F-FDG PET-CT and temporal artery biopsy, performed within one week of steroid initiation, rendered GCA unlikely, in line with recent data suggesting that diagnostic positivity of PET-CT and temporal artery biopsy may persist up to one week after steroid commencement [14,15]. In this case, diagnostic temporal artery ultrasound for GCA was not performed [16].

HBOT may act as a temporizing measure to sustain retinal viability while spontaneous or therapeutic reperfusion occurs [17]. It is postulated to improve oxygen delivery to ischemic inner retinal layers by enhancing diffusion from the choroidal circulation, which remains patent in CRAO [18]. However, the therapeutic window is narrow, with evidence supporting initiation ideally within 6-12 hours [17]. In this case, delays in transport of the patient to a tertiary referral center likely limited the benefit. This underscores the logistical and temporal challenges of CRAO management, particularly in facilities without immediate hyperbaric capabilities.

The patient’s later retinal neovascularization illustrates the importance of both short- and long-term ophthalmic surveillance. Approximately 18% of CRAO patients develop ocular neovascularization, typically within 8-10 weeks [19], yet optimal follow-up strategies remain undefined [20].

## Conclusions

Simultaneous bilateral CRAO is an exceptionally rare and visually devastating presentation that poses substantial diagnostic and therapeutic challenges. This case highlights the importance of rapid recognition, urgent multidisciplinary stroke-based evaluation, and early time-sensitive intervention. It also illustrates the current uncertainty surrounding optimal management of unilateral or bilateral CRAO. Although systemic thrombolysis, IOP-lowering strategies, corticosteroids, and HBOT may be considered in selected patients, robust evidence supporting their efficacy remains limited, particularly in anticoagulated individuals. The subsequent development of ocular neovascularization further underscores the need for close longitudinal ophthalmic surveillance following CRAO. Collectively, this report emphasizes that, in the absence of standardized treatment guidelines, individualized management guided by clinical context, available resources, and evolving evidence remains essential. Well-designed multicenter randomized trials are urgently required to establish evidence-based therapeutic pathways and improve visual outcomes in this devastating condition.
